# Effects of Altering Mitochondrial Antioxidant Capacity on Molecular and Phenotypic Drivers of Fibrocalcific Aortic Valve Stenosis

**DOI:** 10.3389/fcvm.2021.694881

**Published:** 2021-06-24

**Authors:** Carolyn M. Roos, Bin Zhang, Michael A. Hagler, Grace C. Verzosa, Runqing Huang, Elise A. Oehler, Arman Arghami, Jordan D. Miller

**Affiliations:** ^1^Department of Surgery, Mayo Clinic, Rochester, MN, United States; ^2^Department of Physiology and Biomedical Engineering, Mayo Clinic, Rochester, MN, United States

**Keywords:** MnSOD, mitochondria, fibrocalcific aortic valve disease, calcification, oxidative stress

## Abstract

**Background:** While a small number of studies suggest that oxidative stress has an influential role in fibrocalcific aortic valve disease (FCAVD), the roles of specific antioxidant enzymes in progression of this disease remain poorly understood. Here, we focused on selectively altering mitochondrial-derived oxidative stress—which has been shown to alter progression of a myriad of age-associated diseases—on the progression of molecular and phenotypic drivers of FCAVD.

**Methods:** We generated low-density lipoprotein receptor-deficient, Apolipoprotein B100-only mice (LA) that were either haploinsufficient for MnSOD (*LA-MnSOD*^+/−^) or genetically overexpressing MnSOD (*LA-MnSOD*^*Tg*/0^). After 6 months of Western diet feeding, mice underwent echocardiography to assess valvular and cardiac function and tissues were harvested. Quantitative-RT PCR, immunohistochemistry, and histopathology were used to measure changes in molecular pathways related to oxidative stress, calcification, and fibrosis.

**Results:** While reductions in MnSOD increased oxidative stress, there was not an overt phenotypic effect of MnSOD deficiency on valvular and cardiac function in *LA-MnSOD*^+/−^ mice. While markers of canonical bone morphogenetic protein signaling tended to increase in valve tissue from *LA-MnSOD*^+/−^ (e.g., p-SMAD1/5/8 and osterix), we did not observe statistically significant increases in osteogenic signaling. We did, however, observe highly significant reductions in expression of osteopontin, which were associated with significant increases in calcium burden in *LA-MnSOD*^+/−^ mice. Reciprocally, genetically increasing MnSOD did not preserve valve function in *LA-MnSOD*^*Tg*/0^, but we did observe slight reductions in p-SMAD1/5/8 levels compared to their *non*-transgenic littermates. Interestingly, overexpression of MnSOD dramatically increased expression of osteopontin in valve tissue from *LA-MnSOD*^*Tg*/0^ mice, but was not sufficient to attenuate calcium burden when compared to their *LA-MnSOD*^0/0^ littermates.

**Conclusions:** Collectively, this study demonstrates that maintenance of mitochondrial antioxidant capacity is important in preventing accelerated disease progression in a mouse model of FCAVD, but that effectively altering mitochondrial antioxidant capacity as a monotherapeutic approach to slow key histopathological and molecular drivers of FCAVD remains biologically and therapeutically challenging.

## Introduction

Approximately 2–3% of the population over the age of 65 develops fibrocalcific aortic valve disease (FCAVD). While major risk factors for development of FCAVD—such as increasing age and hypercholesterolemia—are frequently shared with atherosclerosis (1), trials of anti-atherosclerotic interventions have yielded largely negative results in patients with FCAVD (2, 3). Consequently, significant gaps remain in our understanding of molecular mechanisms regulating ectopic calcification in the aortic valve.

A large body of work has highlighted the importance of oxidative stress in various pathophysiological processes including tissue remodeling, fibrosis, and calcification. For example, increased oxidative stress contributes to vascular stiffening and remodeling (4, 5) in hypertensive mice, and increases in H_2_O_2_ levels augment osteogenic signaling and increase calcification in atherosclerotic blood vessels (6).

In tissue from humans with end-stage FCAVD, increases in oxidative stress and profound reductions in antioxidant enzyme activity in diseased regions of the valve suggested a strong association between increases in oxidative stress and worsening histopathological features of disease (7). While administration of *non*-selective antioxidants has shown preliminary therapeutic efficacy in preclinical models (8), a causal link between losses in antioxidant capacity in specific subcellular compartments and accelerated ectopic calcification in the valve, however, has been lacking.

Clinically, loss-of-function mutations in mitochondrial antioxidants (such as manganese superoxide dismutase, or MnSOD) are associated with increased risk of vascular calcification. While some reports suggest that experimentally reducing MnSOD worsens vasomotor function (9), atherosclerosis (10), and vascular stiffness (11) in blood vessels from hyperlipidemic mice, data from otherwise unstressed animals suggest that reductions in MnSOD are relatively well-tolerated in most blood vessels. Recently, a report from our laboratory suggested that in chronologically aged mice—with aging being the strongest risk factor for valve calcification in humans—suggested that MnSOD deficiency is not sufficient to induce robust valvular calcification or stenosis (12). It remains unknown, however, if an increase in mitochondrial-derived oxidative stress augments aortic valve calcification and worsens valvular function in mouse models known to develop functional stenosis.

Therefore, the overarching goal of this work was to test the hypothesis that MnSOD is a major regulator of molecular and histopathological drivers of valve disease in a robust mouse model of FCAVD. Our specific hypotheses were 2-fold: (1) Genetic inactivation of one copy of MnSOD will accelerate progression of aortic valve stenosis through augmented osteogenic signaling, increased valvular calcification, and increased tissue remodeling and fibrosis, and (2) genetic overexpression of MnSOD will attenuate osteogenic signaling and valve calcification, reduce tissue remodeling and fibrosis, and slow progression of aortic valve stenosis.

## Materials and Methods

### Animals

Low-density lipoprotein receptor-deficient (*L), apolipoprotein B*^100/100^ (A) mice (referred to as “LA” mice) were used to recapitulate key aspects of FCAVD observed in humans (13–15). In brief, LA mice were crossed with mice that harbored one inactive copy of manganese superoxide dismutase gene (*MnSOD*^+/−^). After generating founders that were homozygous for LA and heterozygous for MnSOD, *LA-MnSOD*^+/+^ × *LA-MnSOD*^+/−^ mice intercrosses were used as a breeding strategy so littermate-matched mice could be compared whenever possible (notably MnSOD^−/−^ mice are not viable for postnatal studies due to poor viability) (16). Mice with one copy of an MnSOD transgene (*MnSOD*^*Tg*/0^) were crossed to the LA-mouse model (17). Once generation of LA-homozygous mice harboring one copy of the *MnSOD* transgene was achieved, experimental animals were maintained using a *LA-MnSOD*^0/0^ × *LA-MnSOD*^*Tg*/0^ breeding strategy so littermate-matched mice could be compared whenever possible. This resulted in parallel colonies with a minimum of four back-crosses maintained on a mixed background (C57BL/6J and 129S4/SvJae mix), which matches the background strain used for initial characterization of this model (15, 18).

Starting at 2 months of age, experimental mice were fed a Western diet (TD.88137; Harlan Teklad) for 6 months. Mice were housed in a climate-controlled environment with 12-h light/dark cycles and had free access to water and feed. All mice were sacrificed following 6 months of western diet feeding (with all mice consequently being sacrificed at ~8 months of age, see Supplementary Table 1) using an overdose of sodium pentobarbital (≥100 mg/kg), and tissues collected are outlined below. While both sexes were studied in roughly equal proportions, we observed neither substantive nor significant differences in response to genetic alteration of MnSOD between sexes. Consequently, both sexes are reported in aggregated datasets throughout the manuscript. All animal experiments were approved by Mayo Clinic's Institutional Animal Care and Use Committee and comply with National Health Institute Office of Laboratory Animal Welfare's guidelines.

### Echocardiography

Prior to sacrifice, all mice underwent high-resolution echocardiography. *LA-MnSOD*^+/−^ mice were imaged using a GE Vivid 7 ultrasound system, 13-MHz linear probe. *LA-MnSOD*^*Tg*/0^ mice were imaged using a Vevo VisualSonics, 40-MHz linear probe to evaluate aortic valve and cardiac function *via* long- and short-axis views, as described previously (12, 19).

### Gene Expression

All three aortic valve leaflets were carefully excised from the heart and placed in RNA lysis buffer (Invitrogen)/1% β-mercaptoethanol (Sigma). Isolation of RNA was performed using Invitrogen spin-columns. After conversion of RNA to cDNA (VILO reverse transcriptase, Invitrogen), quantitative real-time PCR was performed on a StepOnePlus RT-PCR machine. TaqMan Gene Expression Assay primers were used to assess mRNA levels for genes of interest. A full table of primers used is provided in the Supplementary Table 2. All genes were normalized by hypoxanthine phosphoribosyltransferase 1 (HPRT1), a commonly used housekeeper gene, and values are expressed *via* the ΔΔCT method.

### Calcification and Fibrosis

The base of the heart was orientated, embedded in OCT, and stored at −80°C until use. Subsequently, samples were sectioned using a Leica CM3050S cryostat to garner 10-micron-thick frozen cross sections of the aortic valve and stored at −80°C until use.

Evaluation of changes in valvular calcification was done using Alizarin Red staining protocol. Adobe Photoshop was utilized to semiquantitatively assess changes in calcium levels of the aortic valve area using well-established methods from our laboratory and others (12, 18, 20). In brief, positive calcium stain (i.e., red punctate areas) was selected and pixel count of stain was determined in Adobe Photoshop. Subsequently, the aortic valve area was measured to normalize to positive calcium stain to report as a calcium ratio. Bright-field microscopy was used to image all sections at 4 × magnification (Olympus).

Evaluation of changes in valvular fibrosis was done using picrosirius red staining. In brief, sections were fixed using 4% paraformaldehyde, rinsed in PBS, and then incubated for 1 h in picrosirius red solution. Following, picrosirius red incubation samples were briefly rinsed in 0.5% acetic acid water, then dehydrated with increasing concentrations of ethanol, then cleared with xylenes, and finally cover slipped with a xylene-containing mounting medium. All sections were imaged using circularized polarizing light at 20 × magnification on a Zeiss ApoTome 2 Axio Imager. Fiji ImageJ (NIH) was utilized to perform analyses of total collagen and collagen thickness as previously described (21).

### Immunohistochemistry

Frozen cross sections of aortic valve embedded in OCT were stained for immunofluorescence as previously described (22). Primary antibodies used were as follows: osterix (1:500, Abcam), phospho-SMAD1/5/8 (1:100, Cell Signaling), phospho-SMAD3 (1:100, Abcam), and 3-nitrotyrosine (1:100, Abcam). For all staining assays, negative controls were used to confirm validity and consistency of the staining pattern. All sections were stained with Alexa Fluor 647™ (produced in donkey and specific to the respective species secondary; all used at 1:500 dilution, Invitrogen) and mounted in Prolong Gold Anti-fade with DAPI (Invitrogen). Subsequently, a Zeiss LSM 780 confocal microscope set at 20 × magnification was used to focus and capture images of the base portions of the aortic valve leaflet. Following image acquisition, sections were analyzed using IMAGEJ software (NIH).

### Statistical Analyses

All data are expressed in mean ± SEM. Unpaired *T*-tests were used to detect differences between groups at each time point. Where appropriate, corrections for multiple comparisons between groups were made using the Bonferroni method.

## Results

### Does Inactivating One Copy of MnSOD Accelerate Valvular and Cardiac Dysfunction?

**Figure 1:**
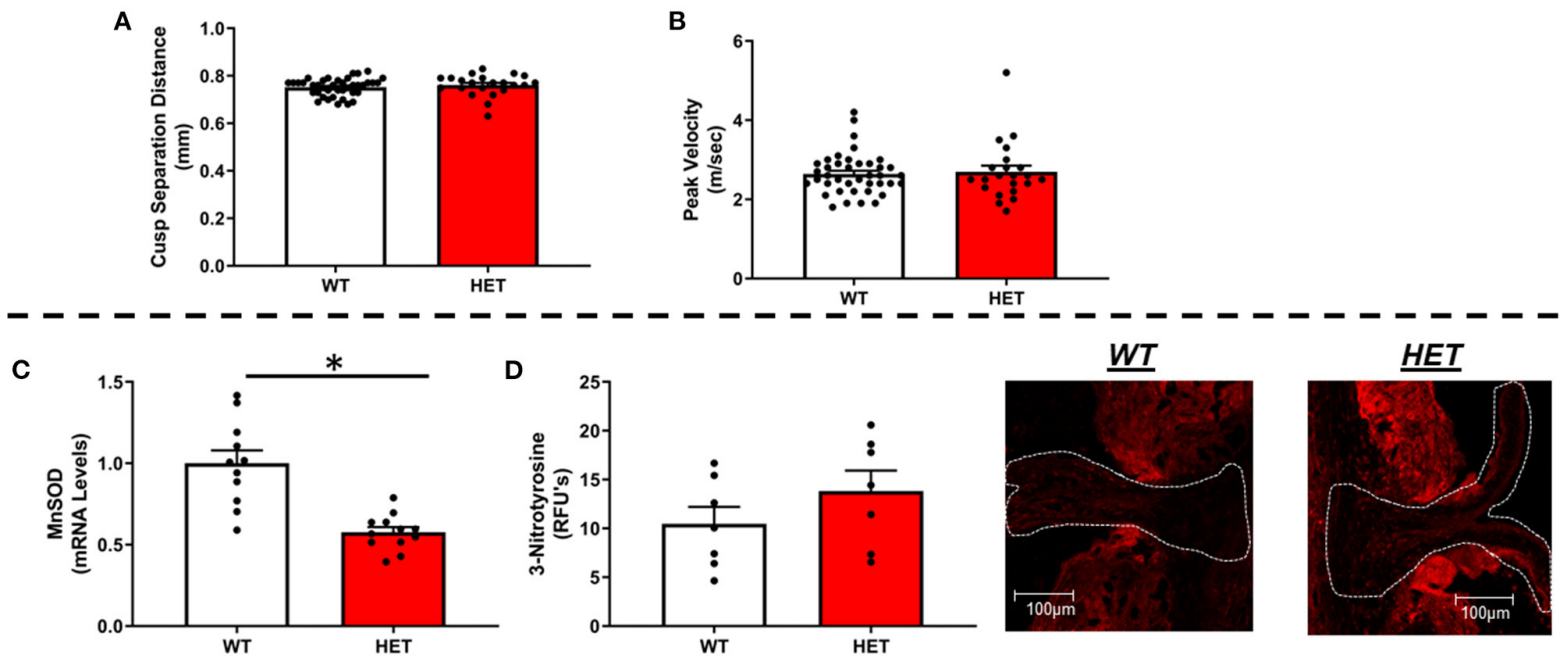
Effects of MnSOD deficiency on valvular function and valvular oxidative stress in hyperlipidemic mice. **(A,B)** Reductions in mitochondrial antioxidant capacity have no effect on cusp separation distance or transvalvular peak velocity compared to wild-type littermates (*n* = 40, 22). **(C)** As expected, MnSOD expression levels were reduced by ~50% in the aortic valve of *LA-MnSOD*^+/−^ mice compared to their wild-type littermates (*n* = 11, 12). **(D)** Losses of mitochondrial antioxidant capacity tended in to increase 3-nitrotyrosine levels—a marker of oxidative stress—compared to wild-type littermates (*n* = 8, 8). For all panels, * denotes *p* < 0.05.

Using echocardiographic measures of cusp separation distance or transvalvular peak velocity, we found that MnSOD haploinsufficiency did not worsen valvular function (Figures 1A,B). Furthermore, neither ejection fraction nor other measures of left ventricular systolic function differed between genotypes (Supplementary Figures 1A,B), nor did cardiac tissue wet weights differ between groups (Supplementary Figure 1C).

### Does Inactivating One Copy of MnSOD Effectively Alter Redox State and Impact Expression of Other Anti- or Pro-oxidant Enzymes in FCAVD?

As expected, there was an observed ~50% reduction in gene expression of MnSOD in heterozygous mice compared to their wild-type littermates (Figure 1C), which was associated with a tendency toward increases in 3-nitrotyrosine levels in the valve cusp (Figure 1D). Consistent with previous reports using this model, expression of CuZnSOD—the other major intracellular SOD isoform—did not differ between genotypes (Supplementary Figure 2A). Extracellular SOD, however, was significantly but modestly reduced in *LA-MnSOD*^+/−^ mice compared to their wild-type littermates (Supplementary Figure 2B). Changes in mRNA levels of other pro-oxidant markers known to play significant roles in cardiovascular tissues (e.g., NOX1, NOX2, and NOX4) were unremarkable and not significant (Supplementary Figures 2C–E).

### Do Reductions in Mitochondrial Antioxidant Capacity Upregulate Pro-osteogenic Pathways and Increase Calcium Burden in FCAVD?

**Figure 2:**
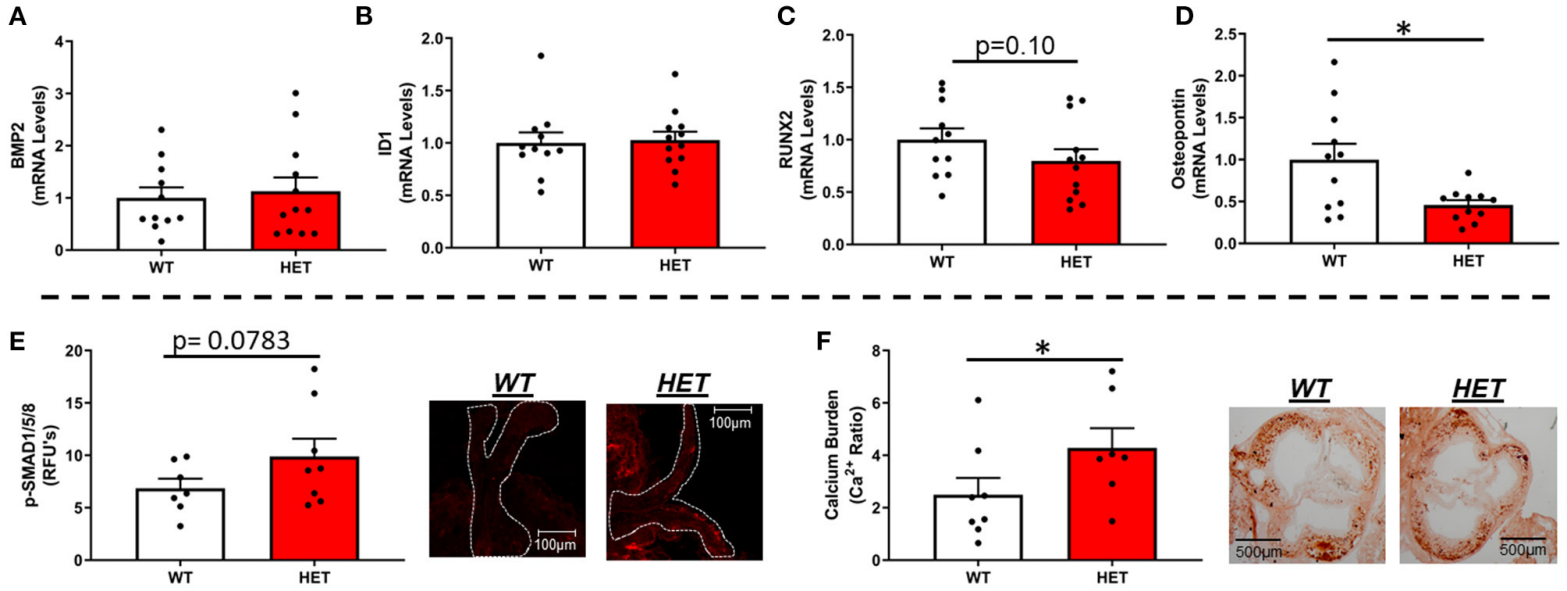
Effects of MnSOD deficiency on valvular osteogenic signaling and calcium burden in hyperlipidemic mice. **(A)** Bone morphogenetic protein-2 (BMP2) expression levels remained unchanged by reductions in mitochondrial antioxidant capacity (*n* = 11, 12). **(B)** Expression levels of ID1—a canonical BMP signaling target—did not change between *LA-MnSOD*^+/+^ and *LA-MnSOD*^+/−^ mice (*n* = 11, 12). **(C)** Expression levels of RUNX2, a BMP-related transcription factor, was modestly reduced in *LA-MnSOD*^+/−^ compared to their *LA-MnSOD*^+/+^ littermates (*n* = 11, 12). **(D)** Interestingly, osteopontin mRNA levels were significantly reduced in aortic valves from *LA-MnSOD*^+/−^ mice compared to *LA-MnSOD*^+/+^ littermates (*n* = 11, 12). **(E)** Immunohistochemical assessment of p-SMAD1/5/8 suggested moderately increased canonical BMP signaling in *LA-MnSOD*^+/−^ mice compared to *LA-MnSOD*^+/+^ mice (*n* = 8, 8). **(F)** Histological evaluations of calcium burden using Alizarin Red staining showed significant increases in valvular calcium burden in *LA-MnSOD*^+/−^ compared to their wild-type littermates (higher magnification images are available in the Supplementary Figure 4) (*n* = 8, 8). For all panels, * denotes *p* < 0.05.

Interestingly, MnSOD haploinsufficiency did not alter mRNA levels of bone-morphogenetic protein-2 (BMP2) (Figure 2A) or BMP4 (Supplementary Figure 3A), although we did observe a tendency toward higher levels of p-smad1/5/8 protein levels in *LA-MnSOD*^+/−^ mice compared to their *LA-MnSOD*^+/+^ littermates (Figure 2E). While expression of other direct BMP target genes was not significantly altered in *LA-MnSOD*^+/−^ mice compared to their *LA-MnSOD*^+/+^ littermates (Figure 2B) and Supplementary Figure 3B), protein levels of osterix—a late-stage marker of osteoblastogenesis—were modestly increased in *LA-MnSOD*^+/−^ mice (Supplementary Figure 3C). Interestingly, while we did observe noticeable and paradoxically unexpected reductions in mRNA levels of the osteogenic transcription factor Runx2 (Figure 2C) in *LA-MnSOD*^+/−^ mice, we also observed substantial and significant reductions in mRNA levels of osteopontin (Figure 2D). Ultimately, these molecular changes resulted in significant increases of calcium burden in *LA-MnSOD*^+/−^ mice compared to their *LA-MnSOD*^+/+^ littermates (assessed using Alizarin red staining in Figure 2F).

### Do Reductions in Mitochondrial Antioxidant Capacity Increase Matrix Remodeling and Fibrogenic Processes in FCAVD?

**Figure 3:**
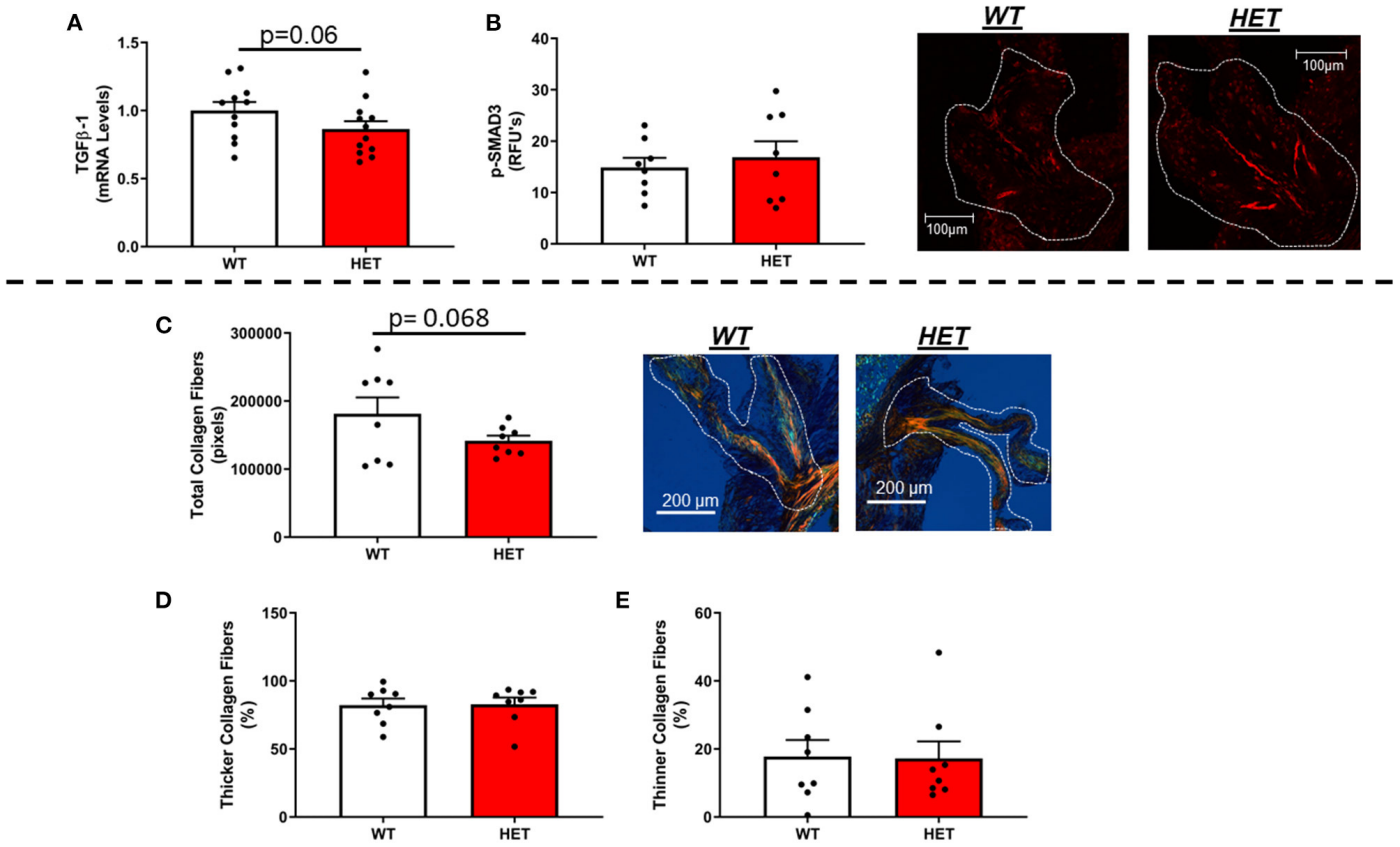
Effects of MnSOD deficiency on valvular fibrogenic signaling, tissue fibrosis, and collagen fiber thickness in hyperlipidemic mice. **(A)** TGFβ-1 expression levels tended to decrease in aortic valves from mice with MnSOD haploinsufficiency compared to their wild-type counterparts (*n* = 11, 12). **(B)** Immunofluorescence of phospho-SMAD3 protein was unchanged with reductions in MnSOD (*n* = 8, 8). **(C–E)** Picrosirius red staining was used to evaluate changes in total collagen burden. **(C)**. Losses of MnSOD in *LA-MnSOD*^+/−^ mice tended to reduce total collagen fibers compared to their *LA-MnSOD*^+/+^ littermates. **(D,E)** Genetic reductions in MnSOD did not alter collagen fiber composition in *LA-MnSOD*^+/−^ mice compared to their *LA-MnSOD*^+/+^ littermates (*n* = 8, 8).

In contrast to our hypothesis, mRNA levels of TGFβ-1—a major regulator of fibrogenesis and matrix remodeling—were unchanged by loss of one copy of MnSOD (Figure 3A). Furthermore, assessment of phospho-SMAD3 proteins levels *via* immunofluorescent staining showed no significant differences between the two genotypes (Figure 3B). Other genes related to fibrosis were largely unchanged as a result of losses in mitochondrial antioxidant capacity in *LA-MnSOD*^+/−^ mice (Supplementary Figures 3D,E). Using picrosirius red staining to assess fibrotic burden and collagen fiber thickness, we found that total fibrotic burden tended to be reduced in *LA-MnSOD*^+/−^ mice compared to wild-type littermates (Figure 3C, *p* = n.s.). There were no observed changes in collagen fiber thickness between genotypes (Figures 3D,E).

### Interim Summary of Effects of Reducing MnSOD on FCAVD

Collectively, genetic inactivation of MnSOD had no effect on valve function, ventricular function, or fibrogenic signaling in this model. Interestingly, despite accelerating accrual of valvular calcium, we observed modest and paradoxically reduced expression of the osteogenic markers Runx2 and Osteopontin in valves from mice with MnSOD haploinsufficiency.

### Does Increasing Mitochondrial Antioxidant Capacity Protect Against Valvular and Cardiac Dysfunction in This Mouse Model of FCAVS?

**Figure 4:**
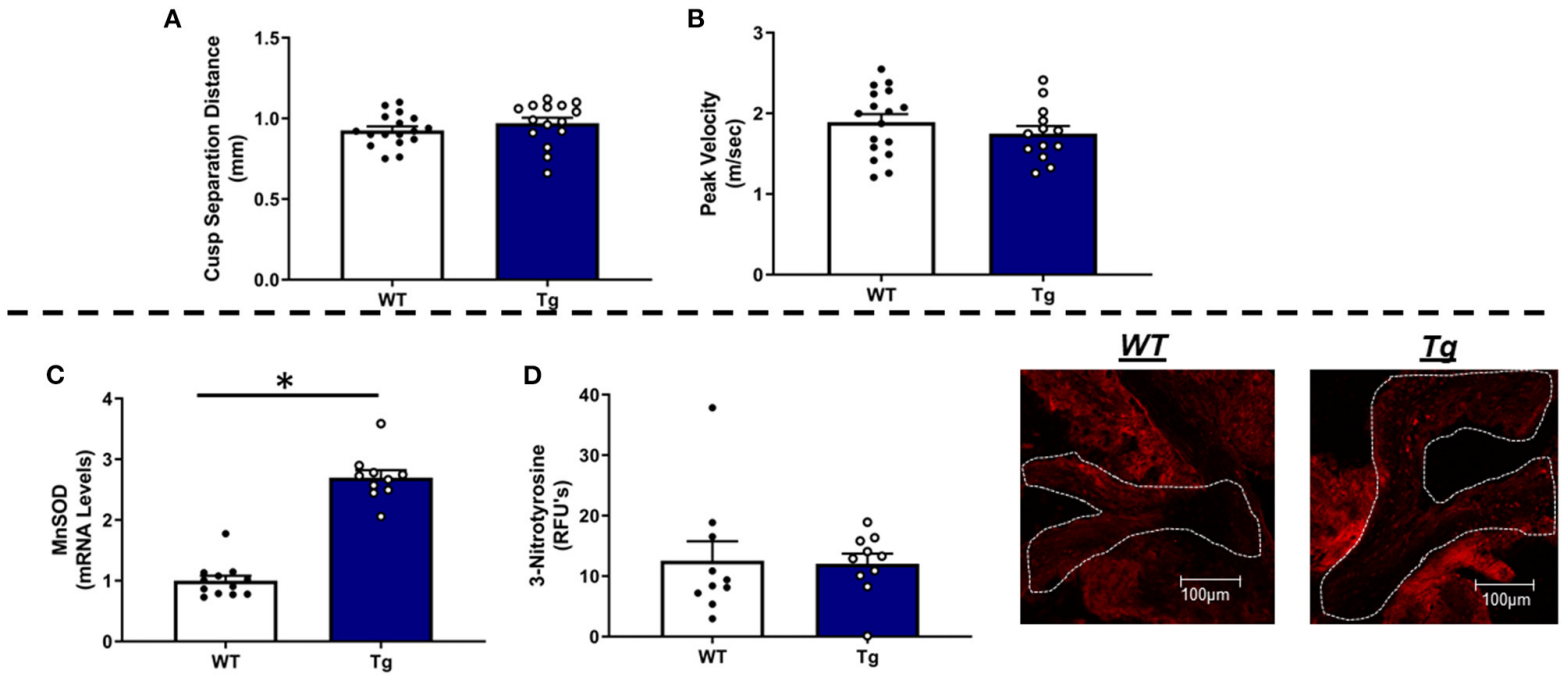
Effects of MnSOD overexpression on valvular function and valvular oxidative stress in hyperlipidemic mice. **(A,B)** Increasing mitochondrial antioxidant capacity had no impact on cusp separation distance and peak velocity compared to *LA-MnSOD*^0/0^ mice (*n* = 17, 15). **(C)** mRNA levels for MnSOD were increased by three-fold in *LA-MnSOD*^*Tg*/0^ mice compared to their wild-type littermates (*n* = 12, 10). **(D)** Interestingly, increasing mitochondrial antioxidant capacity did not reduce 3-nitrotyrosine levels in valves from *LA-MnSOD*^*Tg*/0^ mice compared to their *LA-MnSOD*^0/0^ littermates (*n* = 10, 10). For all panels, * denotes *p* < 0.05.

In contrast to our working hypothesis, transgenic overexpression of MnSOD did not prevent reductions in cusp separation distance, nor did it have an effect on transvalvular peak velocity in *LA-MnSOD*^*Tg*/0^ mice compared to their *LA-MnSOD*^0/0^ littermates (Figures 4A,B). Secondly, increasing mitochondrial antioxidant capacity did not have an overt impact on ejection fraction or left ventricular systolic function (Supplementary Figures 5A,B), nor did cardiac tissue wet weights differ between groups (Supplementary Figure 5C).

### Does Genetically Increasing MnSOD Alter Redox State and Impact Expression of Other Anti- or Pro-oxidant Enzymes in FCAVD?

As expected, MnSOD expression levels were increased approximately three-fold compared to their wild-type littermates (Figure 4C). Surprisingly, levels of 3-nitrotyrosine—indicator of oxidative stress—did not show reductions in valve tissue from mice that carried the MnSOD transgene (Figure 4D). Consistent with prior reports, genetically increasing MnSOD did not result in altered expression of other SOD isoforms in the aortic valve (Supplementary Figures 6A,B), nor did it influence mRNA levels of other pro-oxidant enzymes in aortic valve tissue (Supplementary Figure 6C).

### Does Overexpression of MnSOD Reduce Pro-osteogenic Signaling and Slow Calcium Burden in Hyperlipidemic Mice?

**Figure 5:**
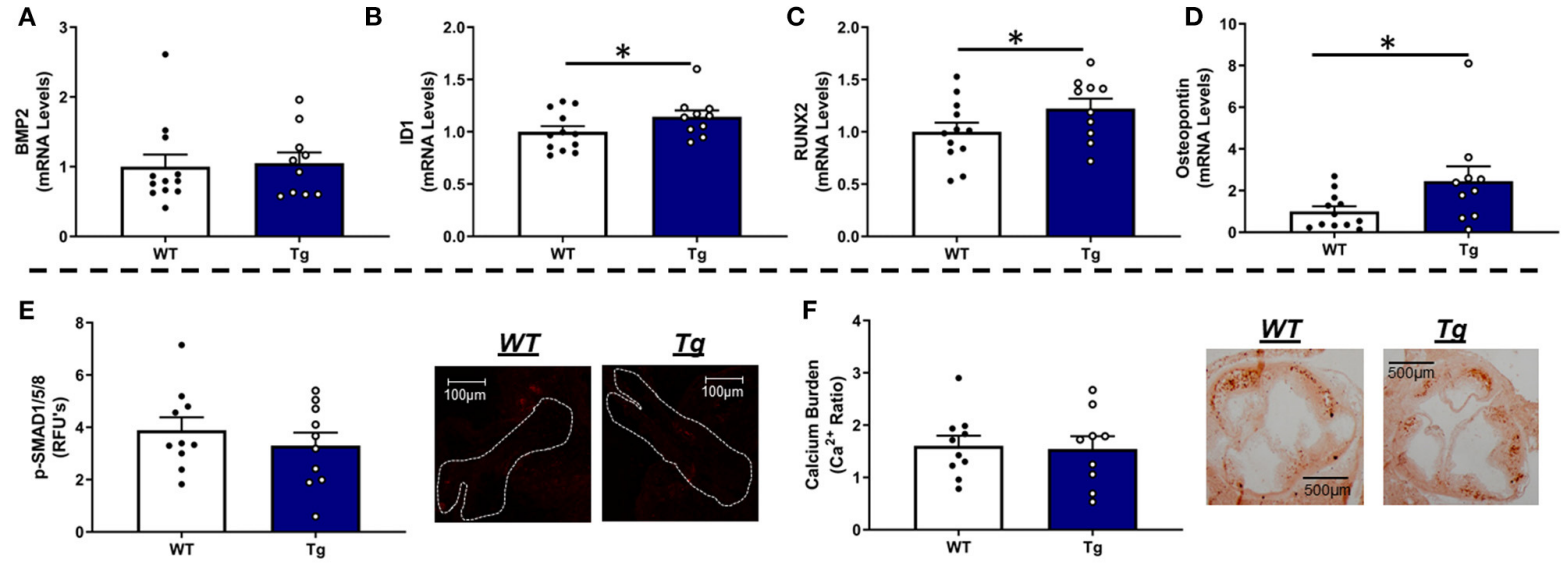
Effects of MnSOD overexpression on osteogenic signaling and calcium burden in valve tissue from hyperlipidemic mice. **(A)** Increasing mitochondrial antioxidant capacity levels did not alter BMP2 expression levels in the aortic valve compared to wild-type littermates (*n* = 12, 10). **(B)** Expression levels of ID1 were significantly increased in *LA-MnSOD*^*Tg*/0^ compared to *LA-MnSOD*^0/0^ littermates (*n* = 12, 10). **(C)** Expression levels of RUNX2 were significantly increased in *LA-MnSOD*^*Tg*/0^ compared to the *LA-MnSOD*^0/0^ littermates (*n* = 12, 10) **(D)** Osteopontin expression levels were significantly increased in LA-MnSOD^Tg/0^ mice compared to *LA-MnSOD*^0/0^ littermates (*n* = 12, 10). **(E)** Increasing MnSOD tended to reduce protein levels of phospho-SMAD1/5/8 in the aortic valve but failed to reach significance (*n* = 10, 10). **(F)** Interestingly, calcium deposition in the aortic valve was unchanged between genotypes despite dramatic increases in MnSOD expression in *LA-MnSOD*^*Tg*/0^ mice (higher-magnification images are available in the Supplementary Figure 8) (*n* = 10, 10). For all panels, * denotes *p* < 0.05.

Consistent with our data in *LA-MnSOD*^+/−^ mice, increasing MnSOD expression in *LA-MnSOD*^*Tg*/0^ mice did not alter mRNA levels of BMP2 or BMP4 (Figure 5A and Supplementary Figure 7A) and only tended to reduce p-SMAD1/5/8 levels in aortic valve tissue (Figure 5E). While expressions of some biomarkers of osteoblastogenesis (such as MSX2) were unchanged (Supplementary Figure 7B), we were surprised to observe significant increases in mRNA levels of the direct p-SMAD1/5/8 target ID1 (Figure 5B) as well as mRNA levels of the osteogenic transcription factor RUNX2 in *LA-MnSOD*^*Tg*/0^ compared to their *LA-MnSOD*^0/0^ littermates (Figure 5C). Interestingly, we again observed a profound effect of MnSOD on mRNA levels of osteopontin, which were now increased in *LA-MnSOD*^*Tg*/0^ mice (Figure 5D). Collectively, these apparently opposing molecular changes resulted in no net impact on calcium burden in *LA-MnSOD*^*Tg*/0^ mice when compared to their *LA-MnSOD*^0/0^ littermates (Figure 5F).

### Does Increasing Mitochondrial Antioxidant Capacity Increase Matrix Remodeling and Fibrogenic Processes in FCAVD?

**Figure 6:**
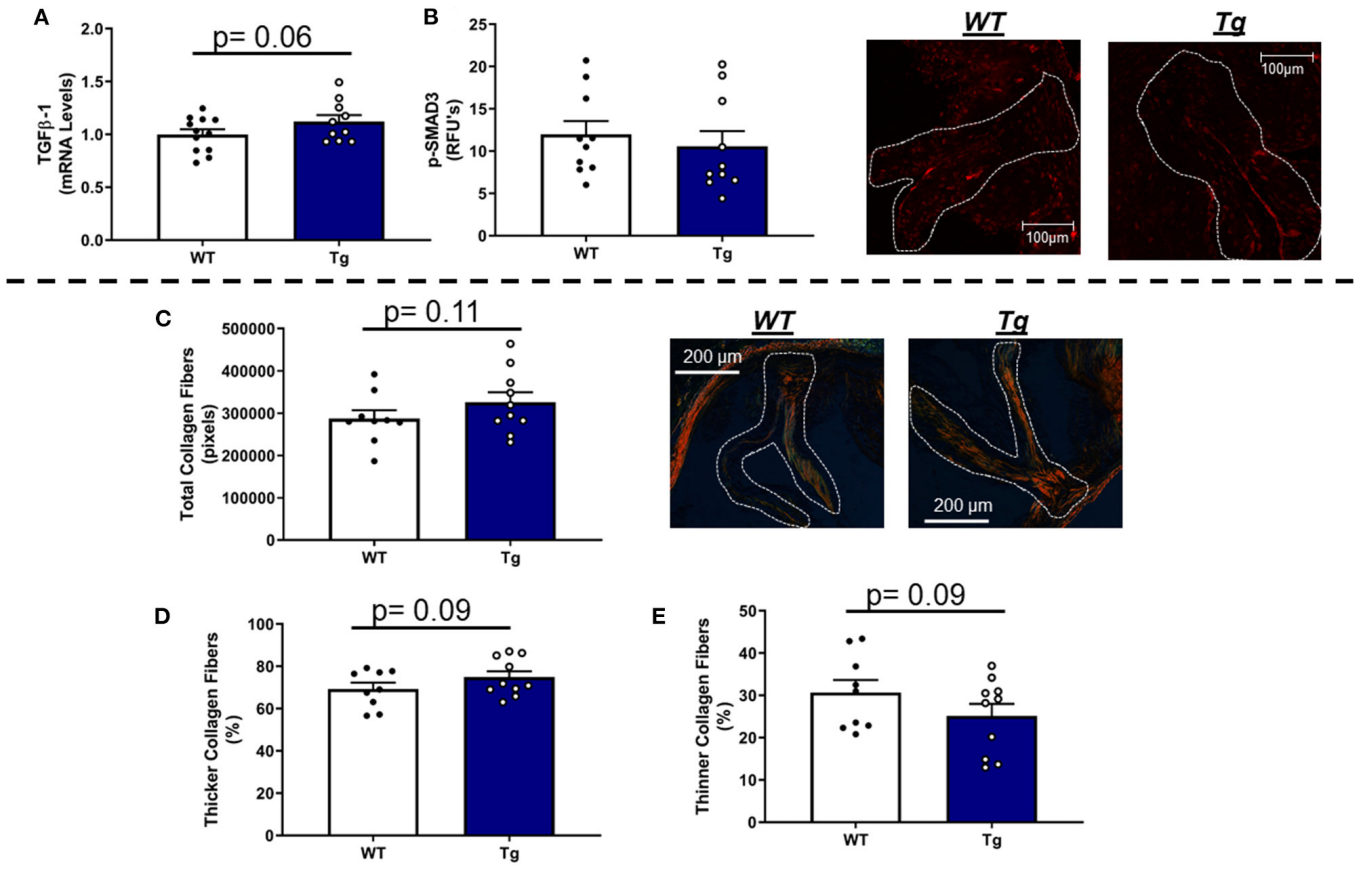
Effects of MnSOD overexpression on fibrogenic signaling and collagen deposition in aortic valves from hyperlipidemic mice. **(A)** Overexpression of MnSOD modestly increased TGFβ-1 expression levels in the aortic valve compared to wild-type mice (*n* = 12, 10). **(B)** Immunofluorescence of phospho-SMAD3 protein was unchanged between *LA-MnSOD*^0/0^ and *LA-MnSOD*^*Tg*/0^ mice (*n* = 10, 10). **(C)** Picrosirius red staining to evaluate changes in collagen burden resulted in modest increases in *LA-MnSOD*^*Tg*/0^ mice compared to wild-type mice (*n* = 10, 10). **(D,E)** The increase in total collagen fibers in *LA-MnSOD*^*Tg*/0^ mice was associated with a tendency toward greater collagen fiber thickness compared to their LA-MnSOD^Tg/0^ littermates (*n* = 10, 10).

In LA mice carrying the MnSOD transgene, mRNA levels of TGFβ-1 were modestly increased (Figure 6A) and associated with parallel changes in COL1A1 mRNA levels (Supplementary Figure 7C). Immunofluorescence-based evaluation of phospho-SMAD3 protein levels in the aortic valve showed no significant differences between *LA-MnSOD*^*Tg*/0^ and *LA-MnSOD*^0/0^ mice (Figure 6B). Histological assessment of tissue fibrosis using picrosirius red staining showed that increasing mitochondrial antioxidant capacity slightly augmented total collagen burden in the aortic valve tissue from *LA-MnSOD*^*Tg*/0^ mice compared to *LA-MnSOD*^0/0^ (Figure 6C). Furthermore, composition analyses of the collagen fibers showed modest reductions in the proportion of thinner collagen fibers in mice overexpressing MnSOD, while thicker collagen fibers were slightly higher in *LA-MnSOD*^*Tg*/0^ mice compared to their LA-MnSOD^0/0^ littermates (Figures 6D,E).

### Interim Summary of Effects of Overexpressing MnSOD on FCAVD

Collectively, genetic overexpression of MnSOD was largely ineffective at reducing biomarkers of oxidative stress in this model and had no beneficial effect on valve function or ventricular function following 6 months of Western diet feeding. While overexpression of MnSOD failed to convey therapeutic benefit with regard to slowing accrual of valvular calcium, we again observed paradoxically increases in expression of the osteogenic markers Runx2 and Osteopontin in valves from mice overexpressing MnSOD and did not observe notable or significant changes in molecular or structural components of extracellular matrix integrity or fibrosis.

## Discussion

The results from this study provide four major novel findings: (1) genetically decreasing or increasing MnSOD did not alter valvular and cardiac function in hyperlipidemic mice; (2) altering MnSOD levels appears to modulate several key molecular changes implicated in progression of FCAVD, although these changes appear to often occur in a conflicting, *non*-orchestrated, and context-dependent manner; (3) the effects of MnSOD on valvular calcification appear to be largely unidirectional, with reductions in MnSOD accelerating valvular calcification but overexpression of MnSOD failing to exert protection against valvular calcification; and (4) ultimately, therapeutic approaches to altering MnSOD levels as a monotherapy remain elusive for the treatment of FCAVD.

### Lifelong Alterations in Mitochondrial Antioxidant Capacity Do Not Affect Aortic Valve Function in Mild-To-Moderate FCAVD

A major finding of this study was that increasing or decreasing expression of MnSOD—over a roughly four-fold range (from deficiency to overexpression)—did not have a detectable impact on aortic valve function this model of FCAVD. This observation is largely consistent with previous reports from our laboratory, suggesting that reductions of MnSOD in aging mice are not sufficient to induce overt valvular stenosis (12), and chronological aging without incremental pathophysiological insults is unlikely to provide a sufficient biological context (e.g., activation of osteogenic signaling) for MnSOD haploinsufficiency to trigger overt calcific valvular disease (12, 23). Importantly, most other models (including hyperlipidemic murine (24), lagomorph (8), or porcine models (25)) do not develop hemodynamically significant aortic valve dysfunction, which consequently precludes the assessment of true therapeutic benefit of antioxidant interventions. As discussed in subsequent sections, however, there is a growing body of literature suggesting that targeting other mechanisms influencing redox state, other specific reactive oxygen species, or developing more “holistic” approaches to improving mitochondrial function and/or redox state may be more effective in modulating pathophysiological cardiovascular tissue calcification and fibrosis and may consequently be more viable to pursue.

### Changes in Mitochondrial Antioxidant Capacity Fail to Orchestrate Changes in Osteogenic Signaling That Are Consistent With Therapeutic Viability

In the current study, we found that p-SMAD1/5/8 levels were coarsely inversely associated with mRNA levels of MnSOD. Our initial focus on BMP signaling stemmed from the seminal reports from Demer and others showing that this signaling cascade is commonly upregulated in cardiovascular lesions and sufficient to result in overt tissue calcification (26). While previous work suggests that BMP signaling can be significantly influenced by reactive oxygen species (and Nox4 activity in particular) (27, 28), our data suggest that the interplay between MnSOD and canonical BMP signaling is unlikely to play a major role in driving key features of FCAVD.

After observing small effect sizes on BMP signaling, we quickly shifted our focus to other major regulators of ectopic osteogenesis such as Runx2, Msx2, and other mechanisms which Chen, Towler, and others have clearly demonstrated is modulated by hydrogen peroxide levels in vascular smooth muscle (6, 29). Here, we observed a unique finding where expression of Runx2 is inversely associated with mRNA levels of MnSOD, and Msx2 is largely unaffected by alterations in mitochondrial redox state. While we were initially surprised by this finding—particularly given the previous work of Liberman demonstrating that systemic administration of *non*-specific antioxidants reduces osteogenic signaling in valve tissue (8)—the changes we observed in Runx2 are entirely consistent with previous reports in orthotopic calcification where increasing MnSOD levels induce Runx2 expression and activity and promote bone ossification (30). Also, the observations from Lai and colleagues stemmed from interventions in cells derived from the aorta (29), where NADPH oxidases are thought to be a larger contributor to increased oxidative stress compared to the diseased valve (7). Collectively, from the perspective of therapeutic utility, one could arrive at the conclusion that MnSOD may function as a “downstream” regulator of a subset of osteogenic mechanisms in valve tissue that is conserved with bone, and would consequently likely to be of limited therapeutic utility.

To determine whether there were other molecular mechanisms that influenced cell and phenotypic fate in FCAVD, we measured the expression of osteopontin and found that it was significantly downregulated in valves from *LA-MnSOD*^+/−^ mice and significantly increased in valves from *LA-MnSOD*^*Tg*/0^ mice. Numerous reports suggest that osteopontin is near-ubiquitously associated with cardiovascular calcification in a variety of models, and the groundbreaking work by Giachelli et al. (31–33) strongly suggests that upregulation of osteopontin is an adaptive and protective response. Importantly, while this may serve to protect against calcific mass expansion in the aortic valve, numerous reports also suggest that upregulation of osteopontin can independently contribute to mitochondrial dysfunction, tissue fibrosis, and cellular apoptosis (34, 35). Ultimately, while it seems biologically plausible that reductions in osteopontin are a primary, permissive event enabling accelerated valvular calcification in MnSOD-deficient mice (or reciprocally, a primary protective mechanism in MnSOD-transgenic mice), such a complicated molecular interplay warrants further investigation.

### Altering MnSOD Expression Has Little Effect on Fibrogenic Signaling or Fibrosis in FCAVD

Emerging data from our group and others strongly suggest that tissue fibrosis may play a significant role in the pathogenesis of FCAVD in both humans and experimental animal models of valve disease. Clinically, women have been shown to have less valvular calcification compared to men at any given severity of stenosis (even after normalizing for body size), suggesting that other factors such as fibrosis may disproportionately contribute to valvular dysfunction in some patient populations (36). Experimentally, Weiss and colleagues have shown that valvular fibrosis can be a primary driver of valvular dysfunction in hyperlipidemic mice with hyperactivation of the renin–angiotensin system (37).

However, regardless of the relative contributions of cusp fibrosis to valvular dysfunction, our data generally suggest that gain or loss of function of MnSOD does not have substantive effects on overall fibrotic burden or collagen fiber thickness in valve tissue from mice in this model system. Importantly—and as noted above—this lack of an effect was observed despite changes in expression of osteopontin and the *well*-documented impact of osteopontin on fibrosis and matrix remodeling in other tissues (38, 39). Our findings differ from several investigations of the role of MnSOD in the regulation of matrix deposition and remodeling in other tissues (e.g., heart or vasculature), where activity of MnSOD appears to inversely associate with cardiac tissue fibrosis (40) or indices of fibrous cap stability in atherosclerotic plaques (41). Thus, further investigation is warranted into the potential differential unique and context-dependent roles osteopontin may play in FCAVD.

### Limitations

One notable limitation of this study is that overexpression of MnSOD did fail to significantly reduce cellular markers of oxidative stress in *LA-MnSOD*^*Tg*/0^ mice compared to their *LA-MnSOD*^0/0^ littermates. While there is a potential for technical reasons for this observation [e.g., 3-NT staining is not specific to the mitochondria (42)], there are ample data demonstrating that MnSOD can undergo a number of posttranslational modifications in disease states [e.g., deacetylation by sirtuin 3 is essential for optimal MnSOD activity (43)]. Furthermore, it is possible that MnSOD expression is already dramatically suppressed in diseased valve tissue (7), which would serve to minimize the observed biological effect size of reducing or increasing MnSOD expression by moderate genetic alterations. Consequently, future investigation into mechanisms regulating “optimal” mitochondrial enzymatic function—both from a reductionist perspective and from a holistic organelle perspective—will be essential for any successful therapeutic intervention targeting mitochondria.

### Summary and Translational Perspectives

Collectively, our data support the intriguing hypothesis that, in FCAVD, mitochondrial antioxidant capacity is endogenously “titrated” to an optimal level to minimize progression of valvular calcification. Data in support of this hypothesis show that reductions of MnSOD accelerates valvular calcification but that overexpression of MnSOD fails to reduce valvular calcification and results in paradoxical increases in key regulators of osteoblastogenesis such as Runx2. While this latter finding echoes the challenges faced by numerous previous investigators aiming to leverage antioxidant therapies to treat cardiovascular diseases (44, 45), it is possible that specific subtypes of patients [e.g., those with loss-of-function mutations in MnSOD (46)] may benefit significantly from targeted antioxidant therapy. Ultimately, we believe a more nuanced and multipronged approach accounting for the highly context-dependent effects of mitochondrial antioxidant capacity will be essential to the development of novel therapeutics aimed at slowing progression of FCAVD.

## Data Availability Statement

The original contributions presented in the study are included in the article/Supplementary Material, further inquiries can be directed to the corresponding author/s.

## Ethics Statement

The animal study was reviewed and approved by Mayo Clinic Institutional Animal Care and Use Committee.

## Author Contributions

CR: conceptualization, methodology, data curation (histology and immunohistochemistry), interpretation, writing—original drafts, and writing—reviewing and editing. BZ: data curation (histology and immunohistochemistry) and writing—reviewing and editing. MH: data curation (qPCR). GV, EO, and RH: data curation (echocardiography). AA: data curation (histology). JM: conceptualization, methodology, funding, interpretation, writing—original drafts, writing—reviewing and editing, and supervision. All authors contributed to the article and approved the submitted version.

## Conflict of Interest

The authors declare that the research was conducted in the absence of any commercial or financial relationships that could be construed as a potential conflict of interest.
