# Supplementary material for: Effects of Altering Mitochondrial Antioxidant Capacity on Molecular and Phenotypic Drivers of Fibrocalcific Aortic Valve Stenosis
Source: Front Cardiovasc Med. 2021 Jun 24;8:694881. doi: 10.3389/fcvm.2021.694881 (PMC8263922; doi:10.3389/fcvm.2021.694881)
Supplement: Supplementary file 1 [file Data_Sheet_1.pdf]

Strain	***Average Time on Diet (months)***	***Average Age at Sacrifice (months)***
LA-MnSOD$^{+/+}$	6.55 ± 0.08	8.46 ± 0.09
LA-MnSOD$^{+/-}$	6.65 ± 0.11	8.67 ± 0.12
LA-MnSOD$^{0/0}$	6.25 ± 0.04	8.44 ± 0.08
LA-MnSOD$^{Tg/0}$	6.30 ± 0.04	8.39 ± 0.07

Supplementary Table 1: *Age and duration of western diet treatment for all experimental animals at time of sacrifice.*

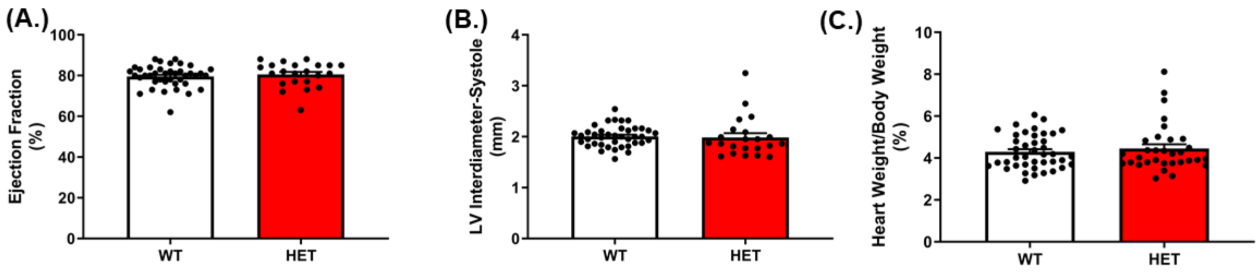

Supplementary Figure 1: Effects of MnSOD-deficiency on cardiac function in hyperlipidemic mice. Panel (A.) Measurement of ejection fraction was unchanged between *LA-MnSOD*$^{+/+}$ and *LA-MnSOD*$^{+/-}$ mice. (n= 40, 22) Panel (B.) Reductions in MnSOD did not alter left ventricular dimensions during systole, suggesting that left ventricular function is well-preserved despite losses in MnSOD. (n= 40, 22) Panel (C.) Heart mass was unaffected by MnSOD-deficiency as demonstrated by measurements of heart wet weight normalized to body weight (n= 41, 32)

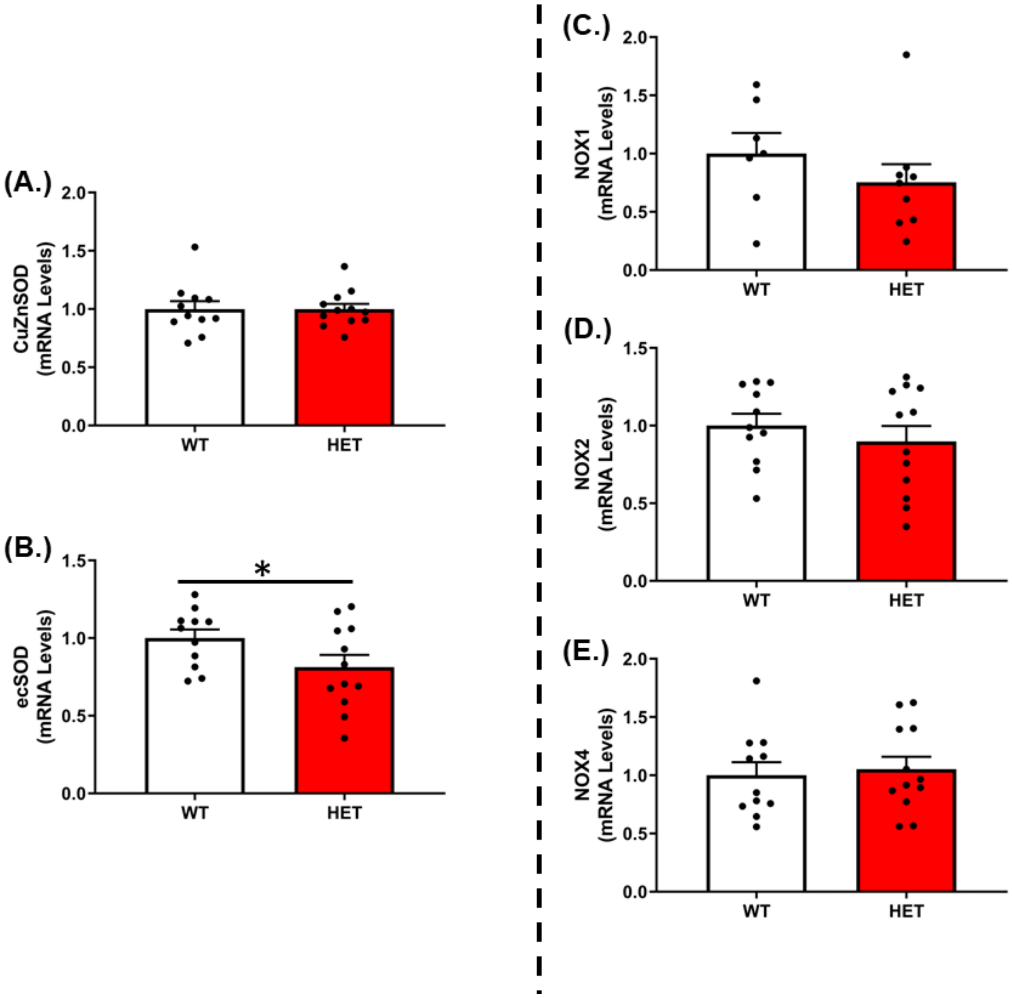

Supplementary Figure 2: Effects of MnSOD-deficiency on other SOD isoforms and NOX isoforms in aortic valves from hyperlipidemic mice. Panel (A.) Copper-zinc SOD (CuZnSOD)—a cytosolic SOD—was not changed with genetic haploinsufficiency of MnSOD. Panel (B.) Interestingly, there was small but significant reduction in ecSOD expression levels in *LA-MnSOD*$^{+/-}$ mice compared to wild-type littermates. Panels (C.-E.) Genetic reductions of MnSOD did not upregulate expression levels of NOX isoforms in the aortic valve (Panel C, n=7, 9; Panels A.-B, D.-E., n= 11, 12) (* denotes p<0.05)

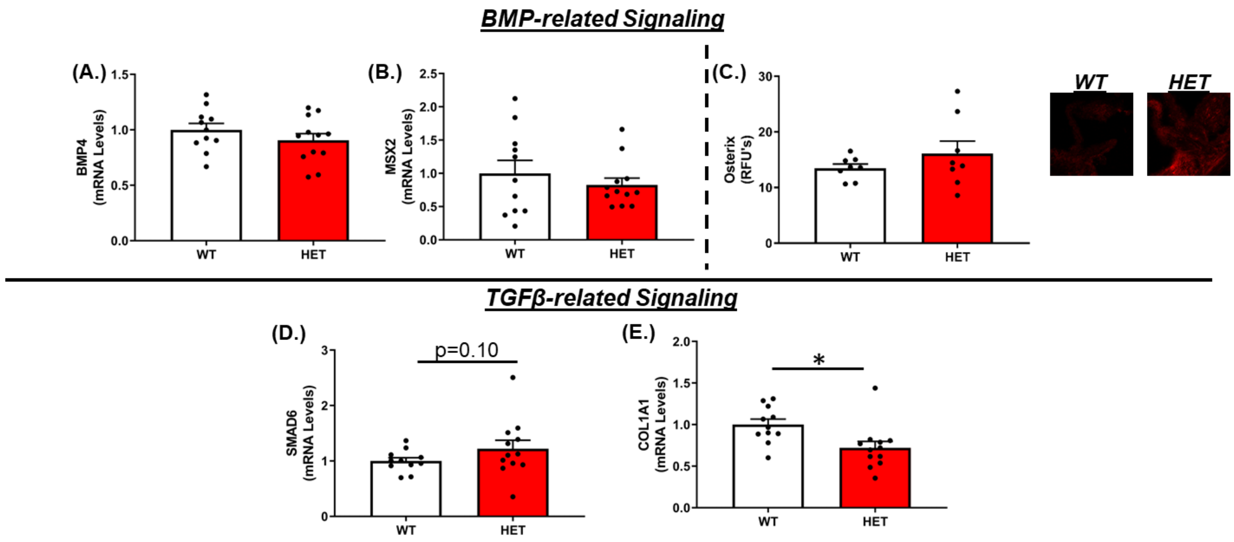

Supplementary Figure 3: Effects of MnSOD-deficiency on pro-osteogenic and pro-fibrotic markers in aortic valves from hyperlipidemic mice. Panels (A.-B.) Reductions of MnSOD did not alter gene expression levels of BMP-related signaling molecules compared to wild-type littermates. (n= 11, 12) Panel (C.) Immunohistochemical assessment of osterix—a late stage osteoblast marker—was modestly increased in *LA-MnSOD*$^{+/-}$ mice compared to their *LA-MnSOD*$^{+/+}$ littermates but failed to reach significance. (n=8, 8) Panels (D.-E). Other key regulators and/or targets of TGFβ were measured. Reductions in MnSOD did not alter SMAD6 expression levels. Surprisingly, however, COL1A1 was significantly reduced in *LA-MnSOD*$^{+/-}$ compared to *LA-MnSOD*$^{+/+}$ littermates despite a lack of effect of MnSOD haploinsufficiency on valvular fibrosis. (n= 11,12) (* denotes $p<0.05$)

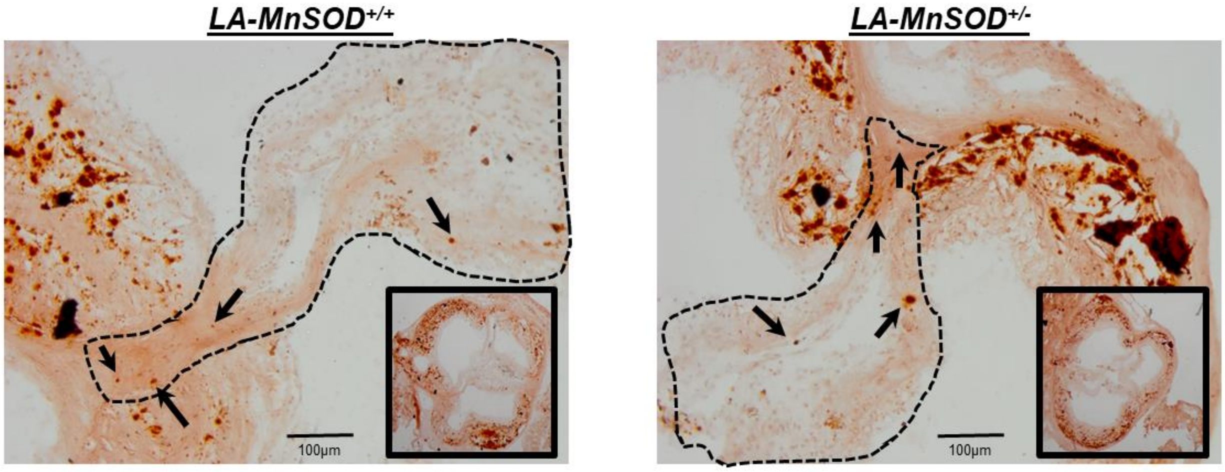

Supplementary Figure 4: Higher magnification images of alizarin red staining in LA-MnSOD$^{+/+}$ and LA-MnSOD$^{+/-}$ mice. Representative images presented in Figure 2 (4x magnification, inset) have been increased to a magnification of 20x. Black arrows highlight positive calcium stain found on the aortic valve cusps.

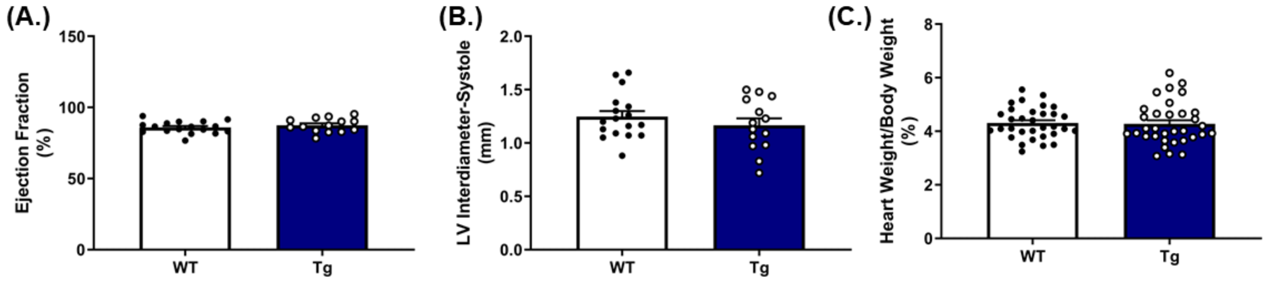

Supplementary Figure 5: Effects of MnSOD-overexpression on cardiac function in hyperlipidemic mice. Panel (A.) Ejection fraction remained similar between transgenic and non-transgenic mice despite substantial increases in MnSOD expression (n= 17, 15) Panel (B.) Left ventricular interdiameter during systole remained the same between *LA-MnSOD*$^{0/0}$ mice and *LA-MnSOD*$^{Tg/0}$ mice, suggesting increasing MnSOD levels yielded no improvement on left ventricular function. (n= 17, 15) Panel (C.) Wet weights of the heart normalized to body weight remained unchanged between genotypes (n= 32, 33)

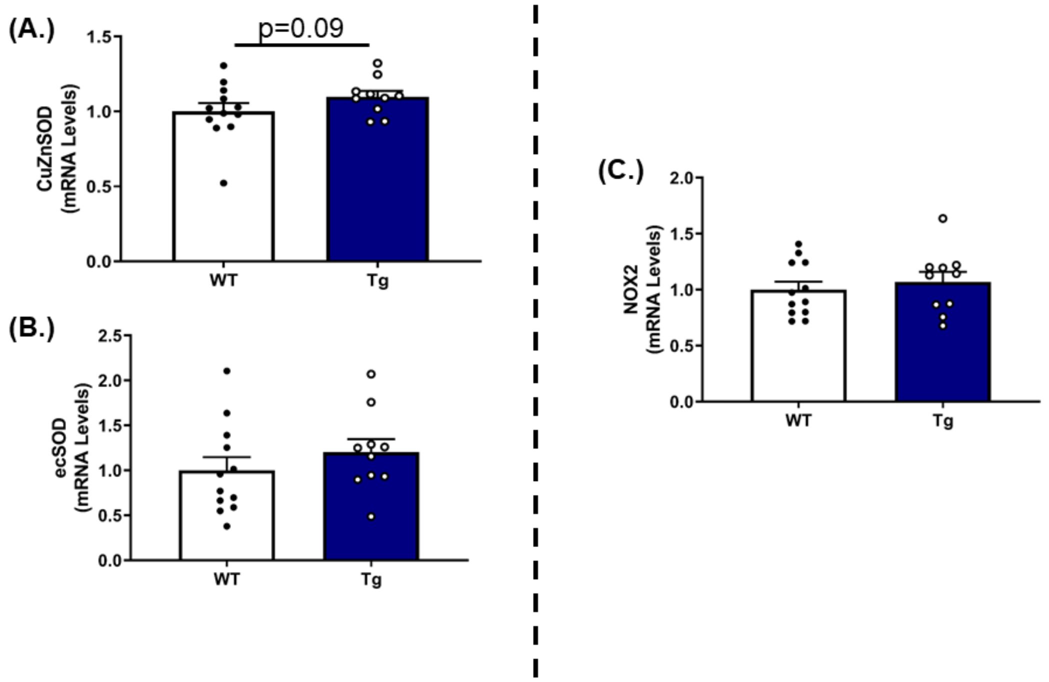

Supplementary Figure 6: Effects of MnSOD-overexpression on expression of other SOD isoforms and pro-oxidant enzyme expression in aortic valves from hyperlipidemic mice. Panels (A.-B.) Transgenic overexpression of MnSOD did not significantly alter expression levels of other SOD isoforms—CuZnSOD and ecSOD—in the aortic valve compared to wild-type littermates. Panel (C.) MnSOD-overexpression did not alter expression of the pro-oxidant enzyme NOX2. (All panels: n= 12, 10)

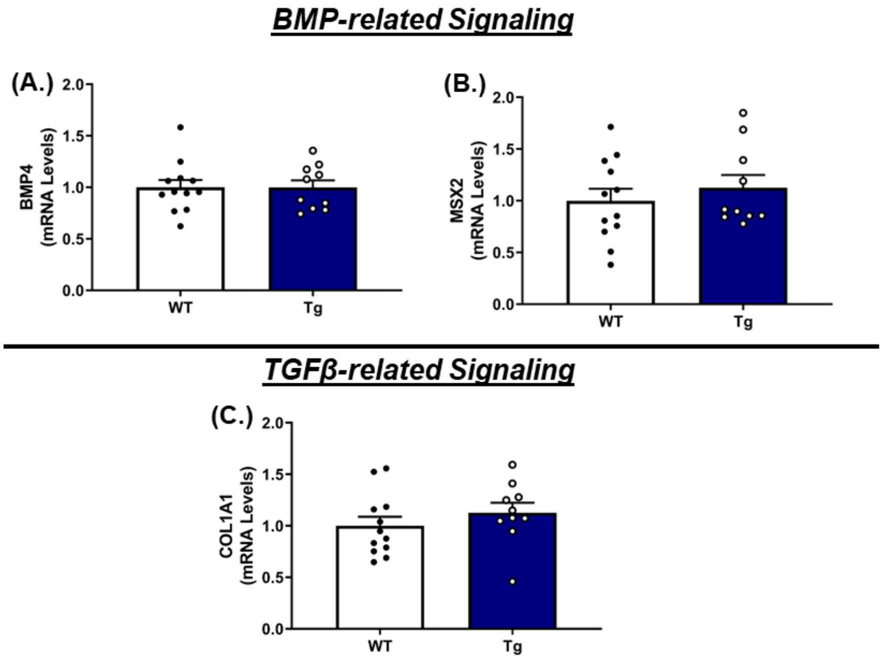

Supplementary Figure 7: Effects of MnSOD-overexpression on pro-osteogenic and pro-fibrotic markers in aortic valves from hyperlipidemic mice. Panels (A.-B.). Consistent with our observations in LA-MnSOD$^{+/-}$ mice, increasing mitochondrial antioxidant capacity did not change expression levels of BMP4 or MSX2. Panel (C.) Expression levels of COL1A1 in aortic valve remained the same between genotypes. (All panels, n= 12, 10)

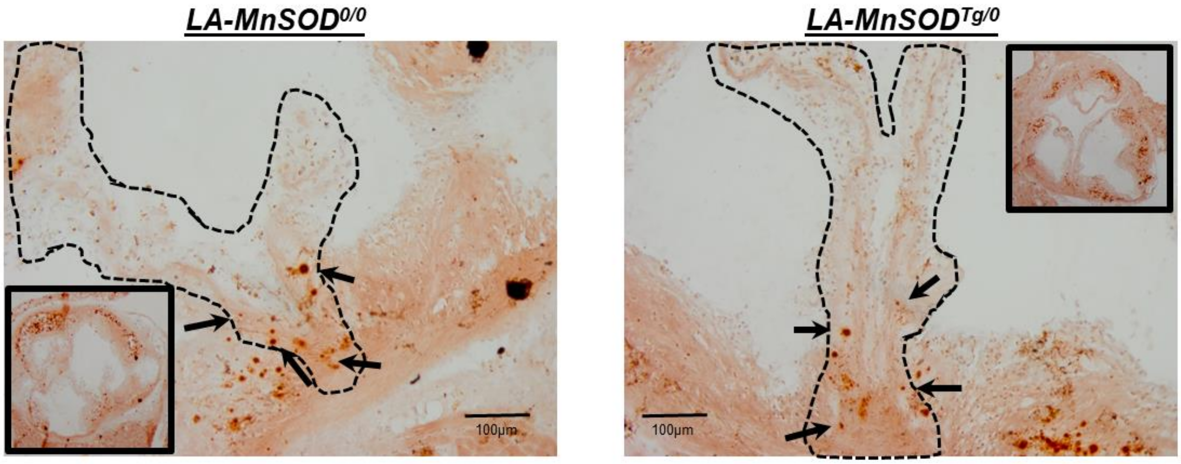

Supplementary Figure 8: Higher magnification images of alizarin red staining in LA-MnSOD$^{0/0}$ and LA-MnSOD$^{Tg/0}$ mice. Representative images presented in Figure 2 (4x magnification, inset) have been increased to a magnification of 20x. Black arrows highlight positive calcium stain found on the aortic valve cusps.
