# Supplementary material for: Effects of Altering Mitochondrial Antioxidant Capacity on Molecular and Phenotypic Drivers of Fibrocalcific Aortic Valve Stenosis
Source: Front Cardiovasc Med. 2021 Jun 24;8:694881. doi: 10.3389/fcvm.2021.694881 (PMC8263922; doi:10.3389/fcvm.2021.694881)
Supplement: Supplementary file 2 [file Data_Sheet_2.docx]

| **Target gene** | **Vendor or Source** | **Primer ID** |
| --- | --- | --- |
| BMP2 | Life Technologies | Mm01340178_m1 |
| BMP4 | Life Technologies | Mm00432087_m1 |
| COL1A1 | Life Technologies | Mm00801666_g1 |
| CuZnSOD | Life Technologies | Mm01344233_g1 |
| ecSOD | Life Technologies | Mm01213380_s1 |
| HPRT | Life Technologies | Mm01545399_m1 |
| ID1 | Life Technologies | Mm00775963_g1 |
| MnSOD | Life Technologies | Mm01313000_m1 |
| MSX2 | Life Technologies | Mm00440330_m1 |
| NOX1 | Life Technologies | Mm00549170_m1 |
| NOX2 | Life Technologies | Mm01287743_m1 |
| NOX4 | Life Technologies | Mm00479246_m1 |
| RUNX2 | Life Technologies | Mm00501584_m1 |
| SMAD6 | Life Technologies | Mm00484738_m1 |
| Spp1 (Osteopontin) | Life Technologies | Mm00436767_m1 |
| TGFβ-1 | Life Technologies | Mm03024053_m1 |

***Supplementary Table 2:*** *List of qPCR primers.*
